# High serum PFAS levels in a population after exposure through drinking water in western Tokyo, Japan and their half-lives estimation

**DOI:** 10.1265/ehpm.25-00330

**Published:** 2026-01-16

**Authors:** Zhaoqing Lyu, Kouji H. Harada, Yoshihiko Sugii, Takenori Ueda, Junko Kimura-Kuroda, Shigeharu Nakachi

**Affiliations:** 1 Kyoto University Graduate School of Medicine; Kyoto Prefectural University, Kyoto, Japan; 2 Honcho Clinic, Kokubunji, Tokyo, Japan; 3 NPO Japan Endocrine-disruptor Preventive Action, Tokyo, Japan; 4 Kumamoto Gakuen University, Chuo-ku, Kumamoto, Japan

**Keywords:** PFAS, Serum, Japanese population, Drinking water, Elimination half-life

## Abstract

**Background:**

Per- and polyfluoroalkyl substances (PFAS) have raised significant health concerns. In 2019, drinking water source was changed due to PFAS contamination in the Tama region, Tokyo, Japan. This study aims to determine the PFAS exposure levels after reduction in drinking water contamination, and to estimate the half-lives of linear isomers of perfluorooctane sulfonate (PFOS), perfluorohexane sulfonate (PFHxS), and perfluorooctanoic acid (PFOA) in serum samples from residents.

**Methods:**

17 participants in 2020 and 2023 from Tama region, Tokyo, Japan (all females, age 53–83 years) were examined. PFAS concentrations in serum in 2023 were measured using gas chromatography-mass spectrometry. Biological half-lives were estimated using first-order kinetics model.

**Results:**

The investigated population was exposed to six PFAS at levels associated with potential health risks, with 95% of them having total PFAS concentrations exceeding 20 ng/mL in 2023. Serum PFOS, PFHxS and PFOA levels decreased from 2000 to 2023 (p < 0.05 by paired *t*-test). The estimated half-lives for PFOS, PFHxS, and PFOA were 3.9 years (95% CI: 3.4–4.6), 5.7 years (95% CI: 4.6–7.5), and 8.0 years (95% CI: 6.0–10.0), respectively. After subtraction of background values in Japan, the estimated half-lives were 2.7 years (95% CI: 2.3–3.4) for PFOS, 5.6 years (95% CI: 4.5–7.4) for PFHxS, and 5.1 years (95% CI: 4.1–6.8) for PFOA.

**Conclusions:**

This study demonstrates participants had still higher serum PFAS levels and these PFAS elimination half-lives in the investigated Japanese population are at years order.

**Supplementary information:**

The online version contains supplementary material available at https://doi.org/10.1265/ehpm.25-00330.

## Graphical Abstract

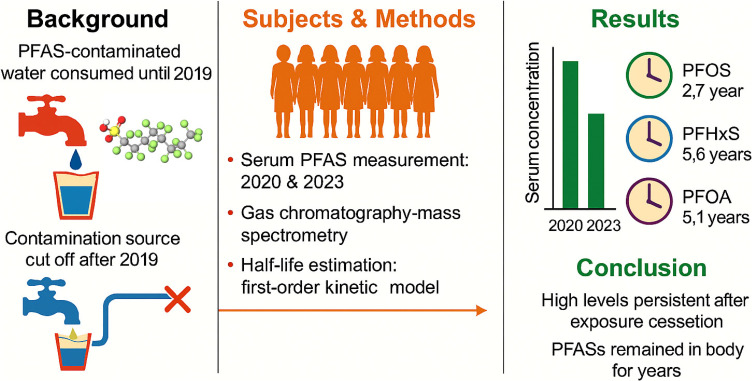

## 1. Introduction

In recent years, concerns over per- and polyfluoroalkyl substances (PFAS) have escalated, largely due to their persistence in the environment and potential risks to human health [1]. Among the most studied PFAS, perfluorooctane sulfonate (PFOS) and perfluorooctanoic acid (PFOA) have been detected globally in various matrices, including human biological samples [2, 3]. Perfluorohexane sulfonate (PFHxS), used as a substitute for PFOS, has also raised health concerns and was added to the Stockholm Convention’s list of regulated chemicals in 2022 [4].

Although animal studies have provided insights into PFAS toxicokinetics [5], the mechanisms underlying PFAS metabolism and elimination in humans remain unclear [6]. Given that chemicals with long biological half-lives tend to bioaccumulate in the body [7], understanding the elimination rates of PFAS in humans is crucial for accurately assessing health risks associated with exposure. Previous research has documented elimination half-lives of PFAS among exposed workers and highly exposed communities in several countries [6]. The estimated mean half-lives ranged from 3.4 to 5.7 years for total PFOS, 1.48 to 5.1 years for PFOA, and 2.84 to 8.5 years for PFHxS [6]. However, data on the Japanese population remain limited.

In the general population, the primary sources of PFAS exposure are diet and consumer products, with drinking water serving as a significant pathway, especially in areas near contamination sources [8, 9]. Numerous cases of PFAS contamination in drinking water have been reported over the past few decades, often linked to industrial sites, military bases, and airports [10]. Japan has experienced similar challenges. A notable case involves the Tama River in the Tokyo region, where PFOS and PFOA contamination was reported as early as the early 2000s [11, 12]. The contamination was traced to the discharge of treated water from a sewage plant handling wastewater from the U.S. Yokota Air Force Base [13]. In 2019–2020, levels of PFOS in the groundwater in the Tama region exceeded the provisional target value for combined PFOS and PFOA (50 ng/L) set by Japanese authorities in 2020 [14, 15]. In response, the Tokyo metropolitan government halted the use of wells with elevated PFAS levels for tap water in the region in June 2019 [16]. Following this action, PFAS concentrations in tap water rapidly dropped to below the provisional target value across the region [17].

In this study, we analyzed serum samples collected in 2020 and 2023 from residents exposed to PFAS-contaminated drinking water in the Tama region, focusing on PFOS, PFOA, and PFHxS. Given the reported half-lives of approximately 3–6 years for major PFAS [6], the three-year sampling interval was considered sufficient to capture meaningful temporal declines in serum concentrations after the cessation of exposure. Our objective was to evaluate PFAS exposure among the study participants following the cessation of prolonged high exposure, and to estimate the elimination half-lives of these PFAS within this exposed group.

## 2. Materials and methods

### 2.1 Sample collection

Originally, human biomonitoring was conducted in August 2020 for residents living in the Tama region, Tokyo, Japan [18]. Blood samples were again collected from identical participants in September 2023, with a time interval of 1099 days. Serum samples were then obtained, and were stored at −30 °C at the Kyoto Human Specimen Bank before measurement [19]. The study was approved by the Ethics Committee of the Kyoto University Graduate School of Medicine (21 July 2021, approval No. R1478). Written informed consent was provided by all participants.

The targeted population consisted of adult residents who had lived and worked in the targeted region for more than 10 years. A total of 19 participants were finally recruited on a voluntary basis, with 18 of them were female. Given that gender and kidney function may affect the excretion of PFAS [20, 21], one male participant and one female participant diagnosed with pyelonephritis at follow-up in 2023 were then excluded from the estimation of half-lives. Since the serum samples collected in 2020 and 2023 were obtained from the same individuals, this matched-pair design helped to account for potential confounding factors, such as differences in lifestyle, that could influence PFAS exposure level. Table 1 shows the characteristics of the 17 participants included in the study. The 17 participants aged from 53 to 83 years (Mean (SD): 70.2 (9.5)), with a mean body mass index (BMI) of 20.7 (SD: 3.4), ranging from 18.1 to 31.3.

**Table 1:** Characteristics of all included participants.

**Characteristic**		**N (%) or mean (SD), median**
Total number		17 (100%)
Sex	Female	17 (100%)
Baseline age (year)		67.2 (9.5), 69.0
Height (cm)		155.7 (5.8), 154.0
Weight (kg)		55.2 (9.0), 55.0
Serum creatinine (mg/dl)		0.68 (0.07), 0.69
eGFR (mL/min/1.73 m^2^)		66.8 (7.7), 66.7

SD, standard deviation; eGFR, estimated glomerular filtration rate.

### 2.2 Determination of chemicals in serum samples

PFAS concentrations in serum were measured using a published method [22]. Concentrations of linear isomers of PFOS, PFOA, and PFHxS in samples collected in 2020 were obtained from the published study. Concentrations of both linear and branched isomers of six PFAS (PFOS, PFHxS, PFOA, perfluorononanoic acid [PFNA], perfluorodecanoic acid [PFDA], perfluoroundecanoic acid [PFUnDA]) in samples collected in 2023 were measured as following procedures [22]. Briefly, 0.4 mL of acetonitrile and 10 ng of surrogate standard were added into 0.1 mL of serum sample. The mixture was centrifuged, and the resulting supernatant was dried. After adding 10 ng of the internal standard, the sample extract was dissolved in a 1% (w/v) bis(4-tert-butylphenyl)iodonium hexafluorophosphate/acetone solution for in-port arylation.

PFAS were then analyzed by gas chromatography-mass spectrometry (Agilent 6890GC-5973MSD) using a DB-5MS capillary column (30 m, 0.25 mm i.d., 0.25 µm film thickness). The injection volume was 1 µL. The full conditions were provided in our previous publication [22]. The limits of detection (LODs) were defined as in the previous publication [22]. The list of analytes and their LODs are summarized in Table S1.

An enzymatic assay using creatinine amidohydrolase was used to determine the plasma creatinine concentration for calculating the estimated glomerular filtration rate (eGFR) (mL/min/1.73 m^2^) values of the participants.

### 2.3 Statistical analysis

#### 2.3.1 Estimation of serum half-life

The elimination rate of PFAS specific to each participant was derived from the decrease of standardized concentration, as shown Equation 1:

$$-k_{i}t=\ln C_{2023}-\ln C_{2020}$$ (1)

*C*_2020_ and *C*_2023_ represent the given PFAS concentrations in an individual participant in 2020 and 2023, respectively; *k_i_* is the rate constant of the first-order reaction derived from an individual participant per day; *t* is the time interval between the two sampling time points, which was 1099 days for all participants.

In the additional analysis, to account for potential background exposure, each PFAS serum concentration were adjusted by subtracting a background value based on data from a national biomonitoring survey in Japan, which was 2.5 ng/mL for PFOS, 1.5 ng/mL for PFOA, and 0.4 ng/mL for PFHxS [23]. The calculation is shown in Equation 2:

$$-k_{s}t=\ln(C_{2023}-C_{0})-\ln(C_{2020}-C_{0})$$ (2)

*C*_0_ represents each background PFAS concentration as descripted above; *k_s_* represents the rate constant of the first-order reaction derived from an individual participant per day after the subtraction of background value.

The point-estimated elimination half-life of a given PFAS was calculated using Equation 3, and the predicted individual elimination half-life of a given PFAS was calculated using Equation 4.

$$t_{1/2}=\frac{\ln2}{\text{mean}(k_{i})}\text{ or }t_{1/2}=\frac{\ln2}{\text{mean}(k_{s})}$$ (3)

$$t_{1/2}=\frac{\ln2}{k_{i}}\text{ or }t_{1/2}=\frac{\ln2}{k_{s}}$$ (4)

The 95% confidence interval (95% CI) for mean *k* was used to calculate the CI for the estimated half-life.

#### 2.3.2 Estimation of kidney function

The estimated glomerular filtration rate (eGFR) (mL/min/1.73 m^2^) was used as an indicator of kidney function of the participants and was calculated based on the participants’ age, sex, and serum creatinine concentrations [24] (Table 1). The association between eGFR and estimated PFAS half-lives was evaluated using simple linear regression analysis.

## 3. Results

### 3.1 Serum PFAS levels in participants

The serum concentrations of linear isomers of PFOS, PFHxS, and PFOA were measured in 2020 and 2023. As shown in Table 2, linear PFHxS had the highest concentrations at both time points, with geometric means (GMs) of 21.0 ng/mL in 2020 and 14.6 ng/mL in 2023. Linear PFOA exhibited the lowest exposure levels among the three compounds, with GMs of 5.5 ng/mL in 2020 and 4.2 ng/mL in 2023. Linear PFOS levels experienced the largest decrease of 40.4% over the two years (p = 0.006 by paired *t*-test). Linear PFHxS concentrations decreased by 29.5% (p = 0.045 by paired *t*-test), while linear PFOA levels declined by 22.6% (p = 0.118 by paired *t*-test). In 2023, serum concentrations of six PFAS, including both linear and branched isomers, were also measured. The distribution of these concentrations is summarized in Table S2. The median level for the sum of the six PFAS was 38.5 ng/mL, ranging from 16.0 to 112 ng/mL. Notably, 95% of participants had total PFAS concentrations exceeding 20 ng/mL, a threshold associated with increased risk of adverse effects according to the National Academies of the United States [25].

**Table 2:** Serum PFAS concentrations (ng/mL) in 17 participants in 2020 and 2023.

	**Sampling year**	**GM**	**Median**	**Min**	**Max**	**Decrease (average, %)**	P*
Linear PFOS	2020	13.9	15.0	6.2	27.0	40.4	0.006
	2023	8.1	8.4	3.3	20.8		
Linear PFHxS	2020	21.0	22.0	5.2	81.0	29.5	0.045
	2023	14.6	14.7	4.1	65.2		
Linear PFOA	2020	5.5	4.9	3.1	12.0	22.6	0.118
	2023	4.2	4.1	2.2	7.9		

GM, geometric means; PFAS, per- and polyfluoroalkyl substance; PFOS, perfluorooctane sulfonate; PFHxS, perfluorohexane sulfonate; PFOA, perfluorooctanoic acid.

*P values by Student’s paired *t*-test for evaluating the difference in log-transformed serum concentrations between sampling years.

BMI did not show a correlation with the serum levels of PFOS, PFHxS or PFOA (p < 0.05 by Pearson correlation analysis). Age was associated only with linear PFOS concentrations at the baseline in 2020 (r = −0.513, p = 0.035 by Pearson correlation analysis).

### 3.2 Half-lives of PFAS and potential contributors

As shown in Table 3, the estimated half-lives for PFOS, PFHxS, and PFOA were 3.9 years (95% CI: 3.4–4.6), 5.7 years (95% CI: 4.6–7.5), and 8.0 years (95% CI: 6.0–10.0), respectively. The mean half-lives for individual participants were 4.6 years (95% CI: 3.6–5.6) for PFOS, 6.5 years (95% CI: 5.6–7.5) for PFHxS, and 9.8 years (95% CI: 7.5–12.1) for PFOA.

**Table 3:** Half-lives for serum PFAS concentrations in 17 participants stratified by age.

	**All (n = 17)**		**<=70 yo (n = 8)**		**>70 yo (n = 9)**		
Estimated half-life (years)^a^							
	Mean	95% CI	Mean	95% CI	Mean	95% CI	
Linear PFOS	3.9	3.4 to 4.6	4.5	3.5 to 6.3	3.5	3.0 to 4.2	
Linear PFHxS	5.7	4.6 to 7.5	6.7	5.8 to 7.9	5.1	3.7 to 8.2	
Linear PFOA	8.0	6.6 to 10.0	8.8	6.8 to 12.4	7.4	5.8 to 10.2	

Individual half-life (years)^b^							
	Mean	95% CI	Mean	95% CI	Mean	95% CI	P*
Linear PFOS	4.6	3.6 to 5.6	5.5	3.7 to 7.3	3.8	3.0 to 4.6	0.119
Linear PFHxS	6.5	5.6 to 7.5	7.0	5.9 to 8.2	6.1	4.8 to 7.5	0.375
Linear PFOA	9.8	7.5 to 12.1	10.5	7.5 to 13.5	9.2	5.9 to 12.6	0.621

95% CI, 95% confidence interval; PFAS, per- and polyfluoroalkyl substance; PFOS, perfluorooctane sulfonate; PFHxS, perfluorohexane sulfonate; PFOA, perfluorooctanoic acid.

^a^Estimated half-lives were calculated from estimates, ln(2)/mean elimination rate (95% CI of the mean).

^b^Individual half-lives (mean and 95% CI) were calculated from samples, ln(2)/individual elimination rate.

*P values for evaluating the difference in individual half-lives between age groups.

To investigate potential contributors to PFAS half-lives, participants were first stratified by age into two groups. As presented in Table 3, participants over 70 years old had shorter half-lives for all three compounds compared to those under 70, though the differences were not statistically significant.

**Fig. 1:**
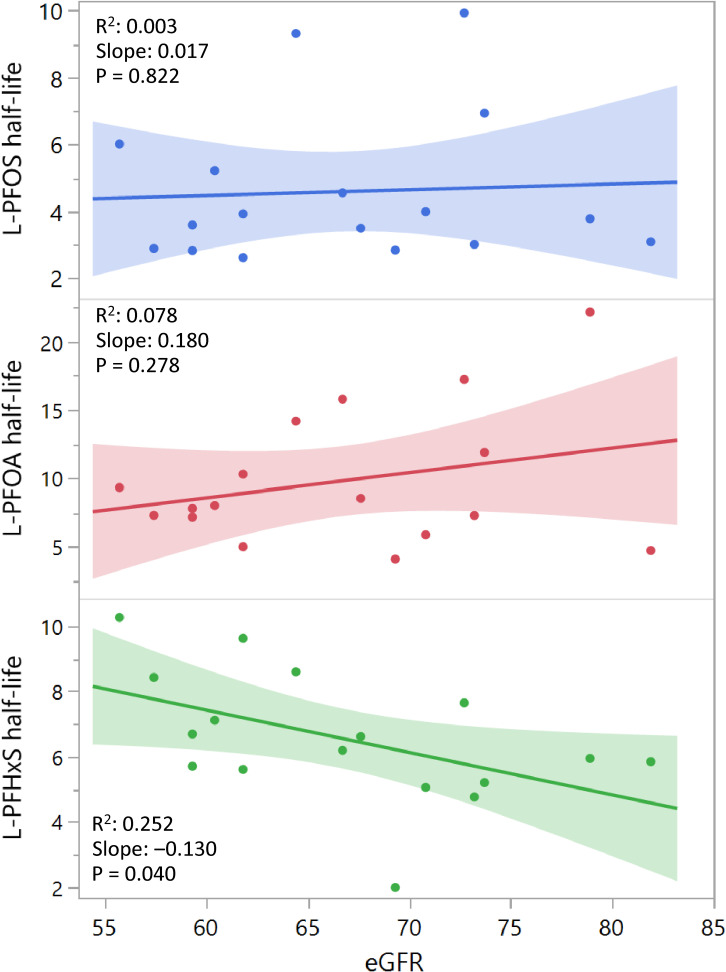
Association between baseline eGFR (mL/min/1.73 m^2^) and half-lives of L-PFOS, L-PFOA, and L-PFHxS. Regression lines with 95% confidence intervals are shown. eGFR: estimated glomerular filtration rate; L-PFOS, linear perfluorooctane sulfonate; L-PFOA, linear perfluorooctanoic acid; L-PFHxS, linear perfluorohexane sulfonate.

Since renal function may also influence the excretion of PFAS [20], we excluded the participant with pyelonephritis as described in *2.1 Sample collection*. Additional investigation was then performed regarding associations between eGFR values of the participants and the individual PFAS half-lives derived in our study. The results are plotted in Fig. 1, which indicates that the eGFR values were only significantly associated with linear PFHxS half-lives in our study (R^2^ = 0.252, p = 0.040).

### 3.3 Influence of background exposure on estimated PFAS half-lives

**Fig. 2:**
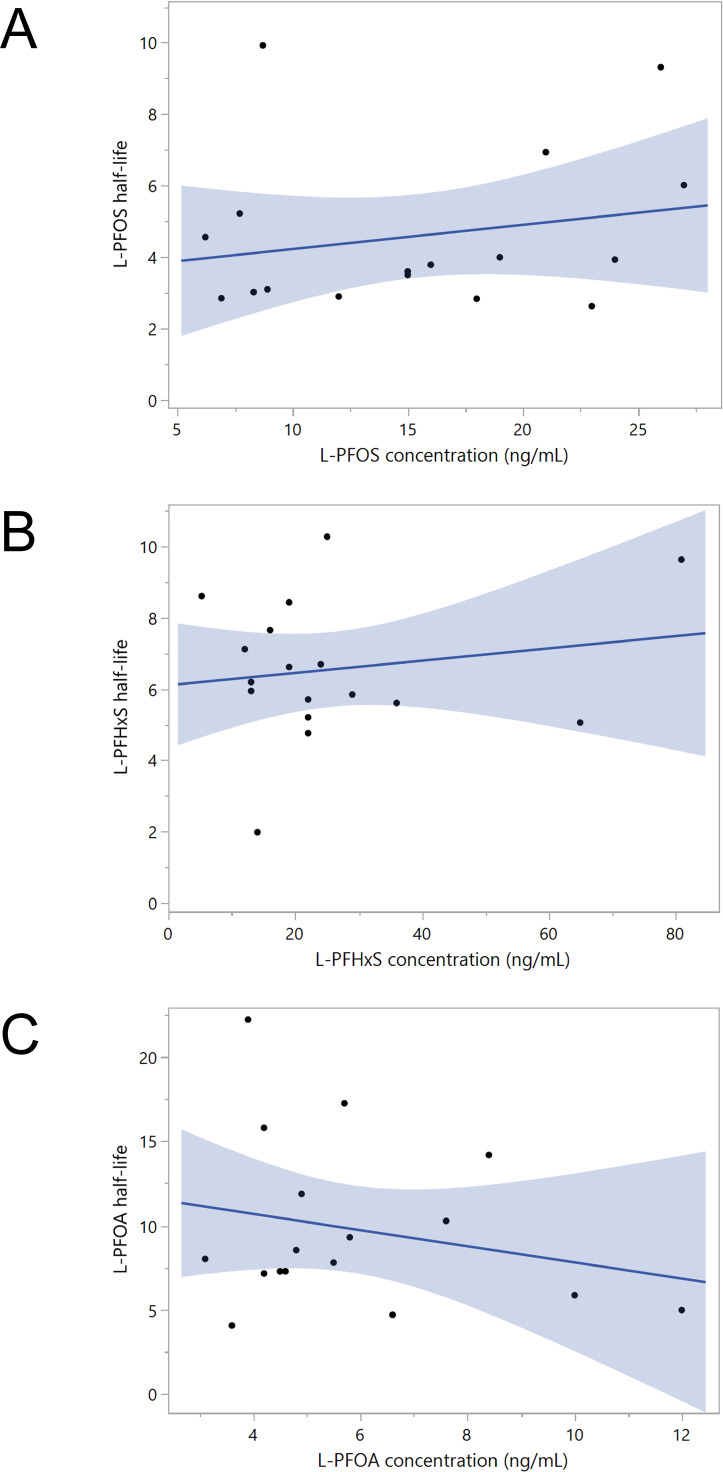
Association between half-lives (year) and serum levels (ng/mL) of L-PFOS (A), L-PFHxS (B), and L-PFOA (C). Regression lines with 95% confidence intervals are shown. Participant number: 17. Half-lives were calculated using the formula ln(2) divided by the individual elimination rate for each participant. L-PFOS, linear perfluorooctane sulfonate; L-PFHxS, linear perfluorohexane sulfonate; L-PFOA, linear perfluorooctanoic acid.

Figure 2 shows the relationships between baseline serum concentrations and predicted individual half-lives of PFOS, PFHxS, and PFOA. Although slight increasing or decreasing trends can be visually observed, no statistically significant correlations were found by Pearson correlation analysis (r = 0.214, p = 0.410 for PFOS, r = 0.168, p = 0.520 for PFHxS, r = −0.231, p = 0.371 for PFOA).

We then stratified participants into high- and low-exposure groups based on their baseline serum PFAS levels (Table 4). For PFOS and PFHxS, participants were categorized based on whether their concentrations were above or below the median levels. Since the PFOA exposure levels were relatively low in this population, we defined the high-exposure group as participants whose baseline PFOA concentrations were higher than the 75th percentile, with the remaining participants classified into the low-exposure group. As shown in Table 4, there was little difference in the estimated and individual half-lives of PFOS and PFHxS between the high- and low-exposure groups. In contrast, PFOA exhibited a 2-year shorter estimated half-life in the high-exposure group, and the mean individual half-life was 2.6 years lower in this group.

**Table 4:** Half-lives for serum PFAS concentrations in 17 participants stratified by baseline exposure level.

	**Low exposure**		**High exposure**		
Number of participants	7 for L-PFOS, 8 for L-PFHxS, 12 for L-PFOA		10 for L-PFOS, 9 for L-PFHxS, 5 for L-PFOA		

Estimated half-life (years)^a^					
	Mean	95% CI	Mean	95% CI	
L-PFOS	3.8	3.0 to 5.0	4.0	3.3 to 5.1	
L-PFHxS	5.4	3.7 to 9.8	6.1	5.3 to 7.2	
L-PFOA^†^	8.7	7.0 to 11.5	6.7	5.0 to 9.9	

Individual half-life (years)^b^					
	Mean	95% CI	Mean	95% CI	P*
L-PFOS	4.5	2.7 to 6.3	4.6	3.4 to 5.9	0.854
L-PFHxS	6.6	5.2 to 7.9	6.5	5.3 to 7.8	0.970
L-PFOA^†^	10.6	7.7 to 13.4	8.0	4.8 to 11.2	0.351

95% CI, 95% confidence interval; PFAS, per- and polyfluoroalkyl substance; L-PFOS, linear perfluorooctane sulfonate; L-PFHxS, linear perfluorohexane sulfonate; L-PFOA, linear perfluorooctanoic acid.

^†^For PFOA, high exposure group consists of participants whose baseline serum concentrations were equal to or higher than the 75th percentile; low exposure group consists of participants whose baseline serum concentrations were below the 75th percentile.

^a^Estimated half-lives were calculated from ln(2)/mean elimination rate.

^b^Individual half-lives were calculated from ln(2)/individual elimination rate.

*P values for evaluating the difference in individual half-lives between exposure level groups.

After the subtraction of background serum PFAS levels, the estimated half-lives were 2.7 years (95% CI: 2.3–3.4) for PFOS, 5.6 years (95% CI: 4.5–7.4) for PFHxS, and 5.1 years (95% CI: 4.1–6.8) for PFOA (Table 5). Notably, the half-life for PFHxS (5.6 years) remained almost unchanged from the pre-adjustment estimate of 5.7 years. In contrast, the half-lives for PFOS and PFOA were shortened by 1.2 years and 2.9 years, respectively, following the background value subtraction.

**Table 5:** Half-lives for serum PFAS concentrations in 17 participants after subtracting background levels.

	**All (n = 17)**		**<=70 yo (n = 8)**		**>70 yo (n = 9)**		
Estimated half-life (years)^a^							
	Mean	95% CI	Mean	95% CI	Mean	95% CI	
Linear PFOS	2.7	2.3 to 3.4	2.8	2.1 to 4.3	2.6	2.2 to 3.3	
Linear PFHxS	5.6	4.5 to 7.4	6.4	5.6 to 7.6	5.0	3.6 to 8.1	
Linear PFOA	5.1	4.1 to 6.8	5.5	4.2 to 8.0	4.9	3.6 to 7.6	

Individual half-life (years)^b^							
	Mean	95% CI	Mean	95% CI	Mean	95% CI	P*
Linear PFOS	3.4	2.5 to 4.3	3.9	2.3 to 5.6	3.0	2.2 to 3.8	0.343
Linear PFHxS	6.4	5.5 to 7.3	6.8	5.7 to 7.9	6.0	4.7 to 7.3	0.643
Linear PFOA	6.6	5.0 to 8.1	7.0	4.6 to 9.4	6.2	4.2 to 8.2	0.435

95% CI, 95% confidence interval; PFAS, per- and polyfluoroalkyl substance; PFOS, perfluorooctane sulfonate; PFHxS, perfluorohexane sulfonate; PFOA, perfluorooctanoic acid.

^a^Estimated half-lives were calculated from ln(2)/mean elimination rate.

^b^Individual half-lives were calculated from ln(2)/individual elimination rate.

*P values for evaluating the difference in individual half-lives between age groups.

## 4. Discussion

### 4.1 PFAS exposure in participants

Due to the contamination identified in drinking water source before 2019 [17], serum PFAS concentrations in the study participants were considerably higher than those reported in the national human biomonitoring survey conducted by the Ministry of the Environment of Japan in 2020, which found mean concentrations of 2.5 ng/mL for PFOS, 1.5 ng/mL for PFOA, and 0.40 ng/mL for PFHxS [23]. Similarly, the Japan Environment, Children’s Study, which investigated over 25,000 mothers between 2011 and 2014, reported median concentrations of 2.90, 0.33, and 1.60 ng/mL for PFOS, PFHxS, and PFOA, respectively [26]. Although our study observed overall declines in serum concentrations of linear PFOS, PFHxS, and PFOA isomers (by 40.4%, 29.5%, and 22.6%, respectively) between 2020 and 2023, the median serum concentration of the six major PFAS (PFOS, PFHxS, PFOA, PFNA, PFDA, and PFUnDA) remained 38.5 ng/mL (16.0–112 ng/mL) in samples collected in 2023, suggesting a continued potential for elevated health risks [25].

In our study, age was negatively associated only with PFOS concentrations at baseline. Previous studies typically report positive associations between PFAS levels and age; however, our participants’ age range (53–83 years) was relatively narrow, reducing variability. The negative association may reflect other age-related factors, such as dietary patterns [27, 28].

### 4.2 Factors influencing PFAS half-lives

Age, kidney function, and menstruation are known determinants of PFAS elimination [20, 29–32]. Because all participants were amenorrheic, this factor did not contribute to variability. Although younger individuals generally display shorter PFAS half-lives [31, 33], our population was ≥50 years, which likely reduced detectable differences between age groups.

The significant association between eGFR and PFHxS half-life supports existing evidence that renal clearance plays a role in PFAS elimination [20]. The lack of association for PFOS and PFOA may reflect the small sample size or limited variability in renal function.

### 4.3 Comparison of serum half-lives with other studies

The estimated half-lives in our study were 2.7 years (95% CI: 2.3–3.4) for PFOS, 5.6 years (95% CI: 4.5–7.4) for PFHxS, and 5.1 years (95% CI: 4.1–6.8) for PFOA after background subtraction (Table 5). The result for PFOS aligns with several previous studies, which reported half-life estimates ranging from approximately 2 to 4 years among populations exposed through contaminated drinking water before exposure cessation [31, 34, 35]. Similarly, consistent with our findings, PFHxS has been reported to have a longer biological half-life than PFOS, with estimates ranging from 4.5 to 15.5 years [31, 33, 34, 36]. A systematic review and meta-analysis by Rosato et al. (2024) [6], which analyzed 13 studies on PFAS half-lives in both highly exposed general populations and workers, found estimated half-lives of 4.7 years (range: 1.7–5.7 years) for PFOS and 5.31 years (range: 2.8–8.5 years) for PFHxS. These estimates are also comparable to our results.

However, our estimated half-life for PFOA (5.1 years after background subtraction) is higher than most previously reported values, which range from 1.7 to 5 years [6, 31–37]. A potential explanation for this discrepancy is the significantly lower PFOA exposure observed among our participants (geometric means of 5.5 ng/mL in 2020 and 4.2 ng/mL in 2023) compared to those in the aforementioned studies (median: 16–691 ng/mL) [31, 32, 34, 36, 37]. Because measured PFOA concentrations in our study were close to background levels, even small unaccounted exposures may have disproportionately influenced the rate constant estimates, biasing the elimination half-life upward. This interpretation is supported by the exposure-stratified analysis, in which participants with higher baseline PFOA levels showed shorter half-life estimates (Table 4). This result suggests that ongoing low-level exposure from other sources may have influenced elimination estimates even after adjustment.

In our analysis of background subtraction, adjusted half-lives for PFOS and PFOA were shorter than the unadjusted values, while PFHxS remained nearly unchanged. This pattern is consistent with pharmacokinetic expectations: when measured concentrations approach the background value (C_0_), subtracting C_0_ increases the apparent elimination rate constant (k_s_), leading to shorter half-life estimates. Because PFHxS concentrations in our study were substantially higher than its reported background level (0.4 ng/mL), the influence of background subtraction was minimal. For PFOA, however, exposure stratification showed a 2-year shorter estimated half-life and a 2.6-year lower mean individual half-life in the high-exposure group (Table 4).

Previous data support the possibility of ongoing exposure in the Japanese population. According to a nationwide biomonitoring survey by the Ministry of the Environment of Japan, mean serum concentrations in 2020 were 2.5 ng/mL for PFOS, 1.5 ng/mL for PFOA, and 0.4 ng/mL for PFHxS [23]. The Japan Environment and Children’s Study reported median levels in mothers across 15 regions during 2011–2014 as 2.9 ng/mL for PFOS, 1.6 ng/mL for PFOA, and 0.33 ng/mL for PFHxS [26]. These data suggest that a certain degree of ongoing exposure may have persisted in our study population. Given that PFOA levels in our participants were much lower than those of PFOS and PFHxS, the estimated half-lives for PFOA may have been more susceptible to the influence of background exposure, potentially explaining the shorter half-lives observed in the high-exposure subgroup. Therefore, unaccounted background exposure likely influenced the results even after subtraction of background values.

### 4.4 Limitations

Our study had several limitations. First, because the study participants were women aged over 50 years and the sample size was small, the results may not be generalizable to other populations. In addition, the small sample size contributed to substantial variability in the individual predicted half-lives. Second, although we discussed about previously reported determinants of PFAS half-life including sex, age, and kidney function [31], we did not assess other potential sources of background exposure, such as dietary intake [27] or the use of consumer products [38]. This may have led to biased estimates even if background value was subtracted in the analysis. Third, we were only able to calculate half-lives of three PFAS using point estimates with a time gap of approximately three years. Fourth, branched isomers of PFAS were not quantified in 2020. They may have different biological half-lives. Future research should include continued follow-up and biomonitoring to provide more comprehensive data on PFAS elimination in the Japanese population.

## 5. Conclusions

This study investigated PFAS concentrations in serum samples collected from female residents (age 53–83 years) exposed to PFAS-contaminated drinking water in the Tama region, Tokyo, Japan, and estimated the half-lives of linear isomers of PFOS, PFHxS, and PFOA. The result showed a potential of increased health risks driven by PFAS exposure in the investigated population. The estimated half-lives for linear PFOS, linear PFHxS, and linear PFOA in the study population were 2.7, 5.6, and 5.1 years, respectively, after subtracting the background levels. Our findings for linear PFOS and linear PFHxS align with previous studies, while the longer half-life observed for linear PFOA may have been influenced by possible unaccounted background exposure.

## Abbreviations

PFAS: Per- and polyfluoroalkyl substances
PFOS: Perfluorooctane sulfonate
PFHxS: Perfluorohexane sulfonate
PFOA: Perfluorooctanoic acid
BMI: Body mass index
PFNA: Perfluorononanoic acid
PFDA: Perfluorodecanoic acid
PFUnDA: Perfluoroundecanoic acid
LOD: Limit of detection
GM: Geometric mean
95% CI: 95% Confidence interval

## Supplementary information

**Additional file 1:** **Table S1.** List of analytes and their limit of detection (LODs) and detection frequencies (n = 19). **Table S2.** Distribution of PFAS concentrations (ng/mL) in 19 participants in 2023.

## Declarations

### Animal welfare

N/A.

### Availability of data and materials

The data that support the findings of this study are available from the corresponding author upon reasonable request.
