# Supplementary material for: High serum PFAS levels in a population after exposure through drinking water in western Tokyo, Japan and their half-lives estimation
Source: Environ Health Prev Med. 2026 Jan 16;31:3. doi: 10.1265/ehpm.25-00330 (PMC12834642; doi:10.1265/ehpm.25-00330)
Supplement: Supplementary file 1 — Additional file 1: Table S1. List of analytes and their limit of detection (LODs) and detection frequencies (n = 19). Table S2. Distribution of PFAS concentrations (ng/mL) in 19 participants in 2023. [file ehpm-31-003-s001.docx]

Table S1. List of analytes and their limit of detection (LODs) and detection frequencies (n = 19).

| Abbreviation | Analyte | LOD (ng/L) | Detection frequency (%) |
| --- | --- | --- | --- |
| L-PFHxS | Perfluoro-n-hexane sulfonate | 0.05 | 100.0 |
| 4m-PFHxS | Perfluoro-4-methyl-pentane sulfonate | 0.01 | 94.7 |
| 3m-PFHxS | Perfluoro-3-methyl-pentane sulfonate | 0.01 | 10.5 |
| 2m-PFHxS | Perfluoro-2-methyl-pentane sulfonate | 0.01 | 0 |
| 1m-PFHxS | Perfluoro-1-methyl-pentane sulfonate | 0.01 | 0 |
| L-PFOS | Perfluoro-n-octane sulfonate | 0.15 | 100.0 |
| 1m-PFOS | Perfluoro-1-methyl-heptane sulfonate | 0.02 | 100.0 |
| 2m-PFOS | Perfluoro-2-methyl-heptane sulfonate | 0.04 | 100.0 |
| 3m-PFOS | Perfluoro-3-methyl-heptane sulfonate | 0.05 | 100.0 |
| 4m-PFOS | Perfluoro-4-methyl-heptane sulfonate | 0.07 | 100.0 |
| 5m-PFOS | Perfluoro-5-methyl-heptane sulfonate | 0.07 | 100.0 |
| 6m-PFOS | Perfluoro-6-methyl-heptane sulfonate | 0.1 | 100.0 |
| 3,5dm-PFOS | Perfluoro-3,5-dimethyl-hexane sulfonate | 0.01 | 63.2 |
| 4,5dm-PFOS | Perfluoro-4,5-dimethyl-hexane sulfonate | 0.01 | 57.9 |
| L-PFOA | Perfluorooctanoic acid | 0.01 | 100.0 |
| 6m-PFOA | Perfluoro-6-methyl-heptanoic acid | 0.01 | 89.5 |
| L-PFNA | Perfluorononanoic acid | 0.01 | 100.0 |
| Iso-PFNA | Iso-perfluorononanoic acid | 0.01 | 100.0 |
| L-PFDA | Perfluorodecanoic acid | 0.01 | 100.0 |
| Iso-PFDA | Iso-perfluorodecanoic acid | 0.01 | 84.2 |
| L-PFUnDA | Perfluoroundecanoic acid | 0.02 | 100.0 |
| Iso-PFUnDA | Iso-perfluoroundecanoic acid | 0.02 | 100.0 |

LOD, limit of detection.

Table S2. Distribution of PFAS concentrations (ng/mL) in 19 participants in 2023.

|  | Linear PFOS | 1m-PFOS | 2m-PFOS | 3m-PFOS | 4m-PFOS | 5m-PFOS | 6m-PFOS | 3,5dm-PFOS | 4,5dm-PFOS | Branched PFOS |
| --- | --- | --- | --- | --- | --- | --- | --- | --- | --- | --- |
| Minimum | 3.31 | 0.11 | 0.04 | 0.32 | 0.37 | 0.53 | 0.53 | ND | ND | 2.22 |
| P25 | 5.49 | 0.59 | 0.06 | 0.53 | 0.83 | 1.44 | 1.22 | ND | ND | 5.18 |
| Median | 8.60 | 0.96 | 0.08 | 0.59 | 1.21 | 1.96 | 1.58 | 0.16 | 0.17 | 6.47 |
| P75 | 10.81 | 1.10 | 0.10 | 0.97 | 1.97 | 3.24 | 1.99 | 0.23 | 0.22 | 10.06 |
| Maximum | 20.78 | 1.91 | 0.16 | 1.83 | 5.60 | 6.71 | 2.77 | 0.33 | 0.27 | 17.65 |
| Mean | 9.40 | 0.91 | 0.08 | 0.73 | 1.67 | 2.56 | 1.65 | 0.14 | 0.12 | 7.86 |
| SD | 4.98 | 0.47 | 0.03 | 0.37 | 1.45 | 1.68 | 0.60 | 0.12 | 0.11 | 4.14 |
| Detection frequency | 100% | 100% | 100% | 100% | 100% | 100% | 100% | 63.2% | 57.9% | - |

|  | Linear PFHxS | 4m- PFHxS | 3m- PFHxS | Branched PFHxS |
| --- | --- | --- | --- | --- |
| Minimum | 3.47 | ND | ND | ND |
| P25 | 9.22 | 0.31 | ND | 0.31 |
| Median | 14.73 | 0.39 | ND | 0.39 |
| P75 | 18.93 | 0.55 | ND | 0.55 |
| Maximum | 65.22 | 1.12 | 0.34 | 1.46 |
| Mean | 17.46 | 0.43 | 0.02 | 0.45 |
| SD | 14.57 | 0.25 | 0.08 | 0.31 |
| Detection frequency | 100% | 94.7% | 10.5% | - |

|  | Linear PFOA | 6m- PFOA | Linear PFNA | Iso-PFNA | Linear PFDA | Iso-PFDA | Linear PFUnDA | Iso-PFUnDA |
| --- | --- | --- | --- | --- | --- | --- | --- | --- |
| Minimum | 2.16 | ND | 1.06 | 0.32 | 0.38 | ND | 0.69 | 0.08 |
| P25 | 3.48 | 0.12 | 1.75 | 0.55 | 0.48 | 0.08 | 1.04 | 0.13 |
| Median | 4.00 | 0.14 | 2.35 | 0.62 | 0.64 | 0.11 | 1.50 | 0.24 |
| P75 | 4.84 | 0.20 | 3.08 | 0.67 | 0.96 | 0.14 | 2.28 | 0.30 |
| Maximum | 7.90 | 0.33 | 5.47 | 0.96 | 1.99 | 0.18 | 4.92 | 0.52 |
| Mean | 4.40 | 0.15 | 2.61 | 0.61 | 0.80 | 0.10 | 1.99 | 0.24 |
| SD | 1.60 | 0.08 | 1.25 | 0.15 | 0.43 | 0.05 | 1.34 | 0.12 |
| Detection frequency | 100% | 89.5% | 100% | 100% | 100% | 84.2% | 100% | 100% |

|  | ΣPFOS | ΣPFHxS | ΣPFOA | ΣPFNA | ΣPFDA | ΣPFUnDA | Σ6PFAS |
| --- | --- | --- | --- | --- | --- | --- | --- |
| Minimum | 5.53 | 3.58 | 2.29 | 1.40 | 0.38 | 0.95 | 16.0 |
| P25 | 11.0 | 9.55 | 3.59 | 2.30 | 0.58 | 1.27 | 32.7 |
| Median | 15.4 | 14.9 | 4.00 | 2.98 | 0.78 | 1.77 | 38.5 |
| P75 | 23.7 | 19.4 | 4.99 | 3.75 | 1.10 | 2.49 | 56.1 |
| Maximum | 38.4 | 66.7 | 8.03 | 6.28 | 2.16 | 5.44 | 112 |
| Mean | 17.3 | 17.9 | 4.55 | 3.22 | 0.90 | 2.23 | 46.1 |
| SD | 8.56 | 14.8 | 1.64 | 1.37 | 0.46 | 1.42 | 22.7 |

P25, 25^th^ percentile; P75, 75^th^ percentile; SD, standard deviation; ND, not detected.
